# 2-(2,3,5,6-Tetra­methyl­benzyl­sulfan­yl)pyridine *N*-oxide

**DOI:** 10.1107/S1600536808029747

**Published:** 2008-09-20

**Authors:** B. Ravindran Durai Nayagam, Samuel Robinson Jebas, J. Jebaraj Devadasan, Dieter Schollmeyer

**Affiliations:** a Department of Chemistry, Popes College, Sawyerpuram 628 251, Tamilnadu, India; b Department of Physics, Karunya University, Karunya Nagar, Coimbatore 641 114, India; c Department of Physics, Popes College, Sawyerpuram 628 251, Tamilnadu, India; d Institut für Organische Chemie, Universität Mainz, Duesbergweg 10-14, 55099 Mainz, Germany

## Abstract

In the title compound, C_16_H_19_NOS, the durene ring and the oxopyridyl ring form a dihedral angle of 82.26 (7)°. The crystal structure is stabilized by inter­molecular C—H⋯O hydrogen bonds, weak C—H⋯π inter­actions and π–π inter­actions [centroid–centroid distance of 3.4432 (19) Å], together with intra­molecular S⋯O [2.657 (2) Å] short contacts.

## Related literature

For bond-length data, see: Allen *et al.* (1987 ▶). For biological activities of *N*-oxide derivatives see: Bovin *et al.* (1992 ▶); Katsuyuki *et al.* (1991 ▶). Leonard *et al.* (1955 ▶); Lobana & Bhatia (1989 ▶); Symons & West (1985 ▶). For related literature, see: Jebas *et al. *(2005 ▶); Ravindran Durai Nayagam *et al.* (2008 ▶).
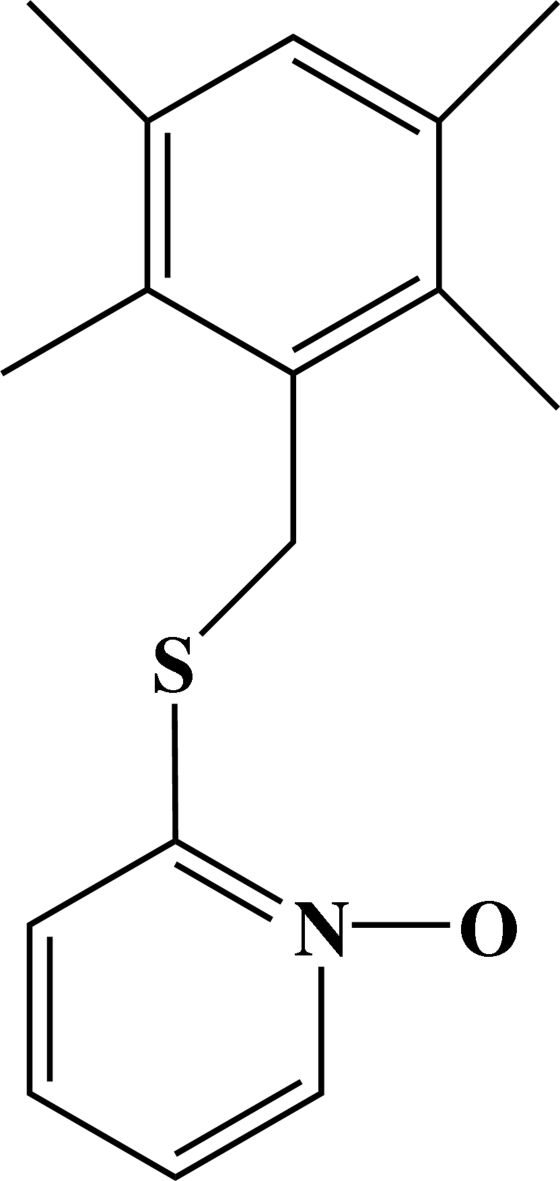


## Experimental

### 

#### Crystal data

C_16_H_19_NOS
*M* *_r_* = 273.38
Monoclinic, 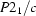

*a* = 16.601 (6) Å
*b* = 9.1562 (8) Å
*c* = 9.696 (4) Å
β = 106.098 (16)°
*V* = 1416.1 (7) Å^3^
*Z* = 4
Cu *K*α radiation
μ = 1.95 mm^−1^
*T* = 193 (2) K
0.51 × 0.38 × 0.03 mm

#### Data collection

Enraf–Nonius CAD-4 diffractometer
Absorption correction: ψ scan (*CORINC*; Dräger & Gattow, 1971 ▶) *T*_min_ = 0.480, *T*_max_ = 0.960
2848 measured reflections
2672 independent reflections
2322 reflections with *I* > 2σ(*I*)
*R*_int_ = 0.064
3 standard reflections frequency: 60 min intensity decay: 2%

#### Refinement

*R*[*F* ^2^ > 2σ(*F* ^2^)] = 0.050
*wR*(*F* ^2^) = 0.144
*S* = 1.05
2672 reflections
176 parameters
H-atom parameters constrained
Δρ_max_ = 0.36 e Å^−3^
Δρ_min_ = −0.34 e Å^−3^

### 

Data collection: *CAD-4 Software* (Enraf–Nonius, 1989 ▶); cell refinement: *CAD-4 Software*; data reduction: *CORINC* (Dräger & Gattow, 1971 ▶); program(s) used to solve structure: *SHELXS97* (Sheldrick, 2008 ▶); program(s) used to refine structure: *SHELXL97* (Sheldrick, 2008 ▶); molecular graphics: *SHELXTL* (Sheldrick, 2008 ▶); software used to prepare material for publication: *SHELXTL* and *PLATON* (Spek, 2003 ▶).

## Supplementary Material

Crystal structure: contains datablocks global, I. DOI: 10.1107/S1600536808029747/sg2262sup1.cif

Structure factors: contains datablocks I. DOI: 10.1107/S1600536808029747/sg2262Isup2.hkl

Additional supplementary materials:  crystallographic information; 3D view; checkCIF report

## Figures and Tables

**Table 1:** Hydrogen-bond geometry (Å, °)

*D*—H⋯*A*	*D*—H	H⋯*A*	*D*⋯*A*	*D*—H⋯*A*
C4—H4⋯O7^i^	0.95	2.51	3.319 (3)	143
C2—H2⋯*Cg*2^ii^	0.95	2.98	3.853 (3)	154

Symmetry codes: (i) 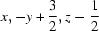; (ii) 
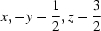
. *Cg*2 is the centroid of the C10–C15 ring.

**Fig. 1.**
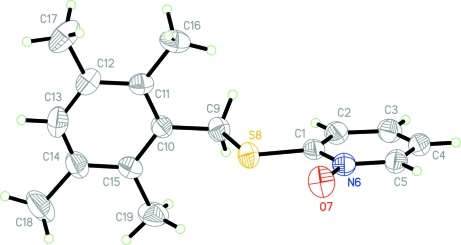
The molecular structure of the title compound, showing 50% probability displacement ellipsoids and the atom numbering scheme.

**Fig. 2.**
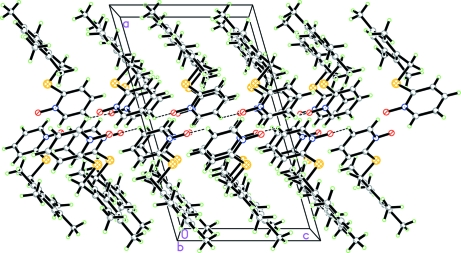
The crystal packing of the title compound, viewed down the b axis.

## supplementary crystallographic information

### Comment

N-Oxides and their derivatives show a broad spectrum of biological activity such as antifungal, antimicrobial and antibacterial activities (Lobana & Bhatia, 1989; Symons *et al.*, 1985). These compounds are also found to be involved in DNA strand scission under physiological conditions (Katsuyuki *et al.*, 1991; Bovin *et al.*, 1992). Pyridine N-oxides bearing a sulfur group in position two display significant antimicrobial activity (Leonard *et al.*, 1955). In view of the importance of N-oxides, we have previously reported the crystal structures of N-oxide derivatives (Jebas *et al.*, 2005; Ravindran Durai Nayagam *et al.*, 2008). As an extension of our work on N-oxide derivatives, we report here the crystal structure of the title compound.

The asymmetric unit of (I) consists of one molecule of 2-(2,3,5,6-Tetramethylbenzylsulfanyl)pyridine N-oxide. The bond lengths and angles agree well with the N-oxide derivatives reported earlier (Jebas *et al.*, 2005) The N—O bond lengths are in good agreement with the mean value of 1.304 (15)Å reported in the literature for pyridine N-oxides (Allen *et al.*,1987).

The pyridine ring and the durene rings are essentially planar with the maximum deviation from planarity being -0.013 (2)Å for atom N6 and -0.011 (2)Å for atom C10 respectively. The dihedral angle formed by the pyridine ring (C1—C5/N6) with the durene ring (C10—C15) is 82.26 (7)°. The atom O7 attached at N6 of the pyridine ring is coplanar, the torsion angle being O7–N6–C5–C4=177.93 (19)°.

The crystal structure is stabilized by intermolecular C—H···O, C–H···π interactions and π–π interactions with the cg1-cg1^i^ distance of 3.4432 (19)Å (*Cg*1:C1—C5/N6) [symmetry code:(i) 1-*X*,1-Y,1-*Z*] together with intramolecular S···O [2.657 (2) Å] short contacts..

### Experimental

A mixture of mono(bromomethyl)durene (0.227 g, 1 mmol) and 1-hydroxypyridine-2-thione sodium salt (0.149,1 mmol) in water (30 ml) and methanol (30 ml) was heated at 333 K with stirring for 30 min. The compound formed was filtered off, and dried. The compound was dissolved in chloroform-methanol (1:1 *v*/*v*) and allowed to undergo slow evaporation. Fine crystals were obtained after a week

### Refinement

After checking for their presence in the Fourier map, all the hydrogen atoms were placed in calculated positions and allowed to ride on their parent atoms with the C—H = 0.95Å (aromatic); C—H = 0.99 Å(methylene) and C—H = 0.98Å (methyl) with *U*_iso_(H) in the range of 1.2U_equ_(C) – 1.5U_equ_(*C*)methyl and methylene.

### Crystal data

**Table:**

C_16_H_19_NOS	*F*(000) = 584
*M**_r_* = 273.38	*D*_x_ = 1.282 Mg m^−^^3^
Monoclinic, *P*2_1_/*c*	Cu *K*α radiation, λ = 1.54178 Å
Hall symbol: -P 2ybc	Cell parameters from 25 reflections
*a* = 16.601 (6) Å	θ = 36–45°
*b* = 9.1562 (8) Å	µ = 1.95 mm^−^^1^
*c* = 9.696 (4) Å	*T* = 193 K
β = 106.098 (16)°	Plate, colourless
*V* = 1416.1 (7) Å^3^	0.51 × 0.38 × 0.03 mm
*Z* = 4	

### Data collection

**Table:**

Enraf–Nonius CAD-4 diffractometer	2322 reflections with *I* > 2σ(*I*)
Radiation source: rotating anode	*R*_int_ = 0.064
graphite	θ_max_ = 69.9°, θ_min_ = 2.8°
ω/2θ scans	*h* = −19→20
Absorption correction: ψ scan (CORINC; Dräger & Gattow, 1971)	*k* = −11→0
*T*_min_ = 0.48, *T*_max_ = 0.96	*l* = −11→0
2848 measured reflections	3 standard reflections every 60 min
2672 independent reflections	intensity decay: 2%

### Refinement

**Table:**

Refinement on *F*^2^	Primary atom site location: structure-invariant direct methods
Least-squares matrix: full	Secondary atom site location: difference Fourier map
*R*[*F*^2^ > 2σ(*F*^2^)] = 0.050	Hydrogen site location: inferred from neighbouring sites
*wR*(*F*^2^) = 0.144	H-atom parameters constrained
*S* = 1.05	*w* = 1/[σ^2^(*F*_o_^2^) + (0.0919*P*)^2^ + 0.3984*P*] where *P* = (*F*_o_^2^ + 2*F*_c_^2^)/3
2672 reflections	(Δ/σ)_max_ < 0.001
176 parameters	Δρ_max_ = 0.36 e Å^−^^3^
0 restraints	Δρ_min_ = −0.34 e Å^−^^3^

### Special details

**Table:**

Geometry. All e.s.d.'s (except the e.s.d. in the dihedral angle between two l.s. planes) are estimated using the full covariance matrix. The cell e.s.d.'s are taken into account individually in the estimation of e.s.d.'s in distances, angles and torsion angles; correlations between e.s.d.'s in cell parameters are only used when they are defined by crystal symmetry. An approximate (isotropic) treatment of cell e.s.d.'s is used for estimating e.s.d.'s involving l.s. planes.
Refinement. Refinement of *F*^2^ against ALL reflections. The weighted *R*-factor *wR* and goodness of fit *S* are based on *F*^2^, conventional *R*-factors *R* are based on *F*, with *F* set to zero for negative *F*^2^. The threshold expression of *F*^2^ > σ(*F*^2^) is used only for calculating *R*-factors(gt) *etc*. and is not relevant to the choice of reflections for refinement. *R*-factors based on *F*^2^ are statistically about twice as large as those based on *F*, and *R*- factors based on ALL data will be even larger.

### Fractional atomic coordinates and isotropic or equivalent isotropic displacement parameters (Å^2^)

**Table:**

	*x*	*y*	*z*	*U*_iso_*/*U*_eq_	
C1	0.37023 (12)	0.4717 (2)	0.5511 (2)	0.0350 (4)	
C2	0.34830 (13)	0.5167 (2)	0.4094 (2)	0.0410 (5)	
H2	0.3054	0.4668	0.3400	0.049*	
C3	0.38878 (15)	0.6342 (3)	0.3689 (3)	0.0482 (6)	
H3	0.3745	0.6647	0.2715	0.058*	
C4	0.45024 (14)	0.7067 (2)	0.4716 (3)	0.0468 (5)	
H4	0.4775	0.7892	0.4456	0.056*	
C5	0.47167 (14)	0.6594 (2)	0.6111 (3)	0.0445 (5)	
H5	0.5141	0.7092	0.6814	0.053*	
N6	0.43286 (11)	0.54200 (19)	0.65006 (19)	0.0382 (4)	
O7	0.45448 (11)	0.49322 (19)	0.78177 (17)	0.0531 (4)	
S8	0.32907 (3)	0.32912 (5)	0.62989 (5)	0.0394 (2)	
C9	0.24486 (13)	0.2652 (2)	0.4783 (2)	0.0392 (5)	
H9A	0.2073	0.3471	0.4354	0.047*	
H9B	0.2681	0.2226	0.4037	0.047*	
C10	0.19752 (13)	0.1513 (2)	0.5361 (2)	0.0351 (4)	
C11	0.13472 (12)	0.1944 (2)	0.5995 (2)	0.0381 (5)	
C12	0.09035 (13)	0.0877 (3)	0.6508 (2)	0.0453 (5)	
C13	0.11026 (15)	−0.0575 (3)	0.6389 (3)	0.0516 (6)	
H13	0.0796	−0.1297	0.6733	0.062*	
C14	0.17289 (15)	−0.1027 (2)	0.5791 (3)	0.0464 (5)	
C15	0.21801 (13)	0.0032 (2)	0.5273 (2)	0.0389 (5)	
C16	0.11488 (16)	0.3533 (3)	0.6159 (3)	0.0528 (6)	
H16A	0.0581	0.3748	0.5559	0.079*	
H16B	0.1553	0.4146	0.5857	0.079*	
H16C	0.1183	0.3736	0.7166	0.079*	
C17	0.02163 (17)	0.1289 (4)	0.7180 (3)	0.0671 (8)	
H17A	−0.0016	0.0403	0.7488	0.101*	
H17B	−0.0228	0.1812	0.6475	0.101*	
H17C	0.0447	0.1920	0.8013	0.101*	
C18	0.1893 (2)	−0.2643 (3)	0.5698 (4)	0.0711 (8)	
H18A	0.1825	−0.2913	0.4694	0.107*	
H18B	0.1494	−0.3197	0.6072	0.107*	
H18C	0.2466	−0.2866	0.6267	0.107*	
C19	0.28812 (17)	−0.0409 (3)	0.4648 (3)	0.0552 (6)	
H19A	0.2969	−0.1467	0.4750	0.083*	
H19B	0.3397	0.0099	0.5158	0.083*	
H19C	0.2733	−0.0147	0.3628	0.083*	

### Atomic displacement parameters (Å^2^)

**Table:**

	*U*^11^	*U*^22^	*U*^33^	*U*^12^	*U*^13^	*U*^23^
C1	0.0376 (10)	0.0298 (9)	0.0427 (11)	0.0045 (8)	0.0200 (8)	−0.0011 (8)
C2	0.0424 (11)	0.0396 (11)	0.0453 (12)	0.0042 (9)	0.0192 (9)	0.0034 (9)
C3	0.0505 (12)	0.0450 (12)	0.0568 (14)	0.0085 (10)	0.0277 (11)	0.0131 (11)
C4	0.0454 (12)	0.0355 (11)	0.0695 (15)	0.0044 (9)	0.0327 (11)	0.0055 (11)
C5	0.0434 (11)	0.0348 (11)	0.0628 (14)	−0.0024 (8)	0.0272 (11)	−0.0073 (10)
N6	0.0417 (9)	0.0343 (9)	0.0433 (9)	0.0019 (7)	0.0198 (8)	−0.0047 (7)
O7	0.0648 (10)	0.0532 (10)	0.0401 (9)	−0.0085 (8)	0.0127 (8)	−0.0019 (7)
S8	0.0464 (3)	0.0368 (3)	0.0370 (3)	−0.0043 (2)	0.0148 (2)	0.00324 (19)
C9	0.0447 (11)	0.0371 (10)	0.0373 (11)	−0.0016 (9)	0.0136 (9)	0.0028 (8)
C10	0.0381 (10)	0.0317 (10)	0.0369 (10)	0.0011 (8)	0.0129 (8)	0.0012 (8)
C11	0.0357 (10)	0.0398 (11)	0.0387 (11)	0.0043 (8)	0.0102 (8)	−0.0032 (9)
C12	0.0383 (11)	0.0557 (13)	0.0437 (12)	−0.0067 (10)	0.0143 (9)	−0.0040 (10)
C13	0.0505 (13)	0.0501 (13)	0.0531 (14)	−0.0177 (10)	0.0128 (11)	0.0048 (11)
C14	0.0513 (13)	0.0323 (11)	0.0504 (13)	−0.0048 (9)	0.0054 (10)	0.0021 (9)
C15	0.0419 (10)	0.0345 (10)	0.0397 (11)	0.0052 (8)	0.0104 (9)	−0.0011 (8)
C16	0.0548 (13)	0.0453 (13)	0.0585 (15)	0.0141 (11)	0.0160 (12)	−0.0079 (11)
C17	0.0470 (13)	0.101 (2)	0.0612 (16)	−0.0133 (14)	0.0274 (12)	−0.0137 (16)
C18	0.084 (2)	0.0320 (12)	0.087 (2)	−0.0009 (12)	0.0070 (17)	0.0041 (13)
C19	0.0612 (14)	0.0486 (13)	0.0611 (15)	0.0143 (11)	0.0256 (12)	−0.0044 (12)

### Geometric parameters (Å, °)

**Table:**

C1—N6	1.365 (3)	C12—C13	1.383 (4)
C1—C2	1.382 (3)	C12—C17	1.510 (3)
C1—S8	1.745 (2)	C13—C14	1.387 (4)
C2—C3	1.382 (3)	C13—H13	0.9500
C2—H2	0.9500	C14—C15	1.401 (3)
C3—C4	1.382 (4)	C14—C18	1.511 (3)
C3—H3	0.9500	C15—C19	1.509 (3)
C4—C5	1.370 (3)	C16—H16A	0.9800
C4—H4	0.9500	C16—H16B	0.9800
C5—N6	1.360 (3)	C16—H16C	0.9800
C5—H5	0.9500	C17—H17A	0.9800
N6—O7	1.306 (2)	C17—H17B	0.9800
S8—C9	1.821 (2)	C17—H17C	0.9800
C9—C10	1.505 (3)	C18—H18A	0.9800
C9—H9A	0.9900	C18—H18B	0.9800
C9—H9B	0.9900	C18—H18C	0.9800
C10—C11	1.406 (3)	C19—H19A	0.9800
C10—C15	1.406 (3)	C19—H19B	0.9800
C11—C12	1.395 (3)	C19—H19C	0.9800
C11—C16	1.510 (3)		
			
N6—C1—C2	119.84 (19)	C12—C13—C14	123.1 (2)
N6—C1—S8	111.06 (15)	C12—C13—H13	118.4
C2—C1—S8	129.10 (17)	C14—C13—H13	118.4
C3—C2—C1	120.0 (2)	C13—C14—C15	118.7 (2)
C3—C2—H2	120.0	C13—C14—C18	119.1 (2)
C1—C2—H2	120.0	C15—C14—C18	122.2 (2)
C2—C3—C4	119.3 (2)	C14—C15—C10	118.9 (2)
C2—C3—H3	120.4	C14—C15—C19	120.5 (2)
C4—C3—H3	120.4	C10—C15—C19	120.6 (2)
C5—C4—C3	119.8 (2)	C11—C16—H16A	109.5
C5—C4—H4	120.1	C11—C16—H16B	109.5
C3—C4—H4	120.1	H16A—C16—H16B	109.5
N6—C5—C4	120.7 (2)	C11—C16—H16C	109.5
N6—C5—H5	119.6	H16A—C16—H16C	109.5
C4—C5—H5	119.6	H16B—C16—H16C	109.5
O7—N6—C5	121.28 (19)	C12—C17—H17A	109.5
O7—N6—C1	118.40 (17)	C12—C17—H17B	109.5
C5—N6—C1	120.32 (19)	H17A—C17—H17B	109.5
C1—S8—C9	101.13 (10)	C12—C17—H17C	109.5
C10—C9—S8	106.60 (14)	H17A—C17—H17C	109.5
C10—C9—H9A	110.4	H17B—C17—H17C	109.5
S8—C9—H9A	110.4	C14—C18—H18A	109.5
C10—C9—H9B	110.4	C14—C18—H18B	109.5
S8—C9—H9B	110.4	H18A—C18—H18B	109.5
H9A—C9—H9B	108.6	C14—C18—H18C	109.5
C11—C10—C15	121.22 (19)	H18A—C18—H18C	109.5
C11—C10—C9	119.71 (19)	H18B—C18—H18C	109.5
C15—C10—C9	119.07 (19)	C15—C19—H19A	109.5
C12—C11—C10	119.2 (2)	C15—C19—H19B	109.5
C12—C11—C16	119.0 (2)	H19A—C19—H19B	109.5
C10—C11—C16	121.7 (2)	C15—C19—H19C	109.5
C13—C12—C11	118.7 (2)	H19A—C19—H19C	109.5
C13—C12—C17	120.2 (2)	H19B—C19—H19C	109.5
C11—C12—C17	121.1 (2)		
			
N6—C1—C2—C3	−1.1 (3)	C15—C10—C11—C16	177.1 (2)
S8—C1—C2—C3	179.56 (16)	C9—C10—C11—C16	−1.9 (3)
C1—C2—C3—C4	−0.9 (3)	C10—C11—C12—C13	0.6 (3)
C2—C3—C4—C5	1.6 (3)	C16—C11—C12—C13	−178.4 (2)
C3—C4—C5—N6	−0.3 (3)	C10—C11—C12—C17	−179.2 (2)
C4—C5—N6—O7	177.93 (19)	C16—C11—C12—C17	1.8 (3)
C4—C5—N6—C1	−1.7 (3)	C11—C12—C13—C14	0.5 (4)
C2—C1—N6—O7	−177.22 (18)	C17—C12—C13—C14	−179.7 (2)
S8—C1—N6—O7	2.2 (2)	C12—C13—C14—C15	−0.4 (4)
C2—C1—N6—C5	2.5 (3)	C12—C13—C14—C18	−179.5 (2)
S8—C1—N6—C5	−178.13 (14)	C13—C14—C15—C10	−0.8 (3)
N6—C1—S8—C9	176.64 (14)	C18—C14—C15—C10	178.2 (2)
C2—C1—S8—C9	−4.0 (2)	C13—C14—C15—C19	178.5 (2)
C1—S8—C9—C10	−173.92 (14)	C18—C14—C15—C19	−2.5 (4)
S8—C9—C10—C11	83.9 (2)	C11—C10—C15—C14	2.0 (3)
S8—C9—C10—C15	−95.2 (2)	C9—C10—C15—C14	−178.98 (19)
C15—C10—C11—C12	−1.9 (3)	C11—C10—C15—C19	−177.3 (2)
C9—C10—C11—C12	179.06 (19)	C9—C10—C15—C19	1.8 (3)

### Hydrogen-bond geometry (Å, °)

**Table:**

*D*—H···*A*	*D*—H	H···*A*	*D*···*A*	*D*—H···*A*
C4—H4···O7^i^	0.95	2.51	3.319 (3)	143.
C2—H2···Cg2^ii^	0.95	2.98	3.853 (3)	154

Symmetry codes: (i) *x*, −*y*+3/2, *z*−1/2; (ii) *x*, −*y*−1/2, *z*−3/2.
